# Point-of-care ultrasound in geriatrics: a national survey of VA medical centers

**DOI:** 10.1186/s12877-023-04313-2

**Published:** 2023-09-27

**Authors:** Maya Gogtay, Ryan S. Choudhury, Jason P. Williams, Michael J. Mader, Kevin J. Murray, Elizabeth K. Haro, Brandy Drum, Edward O’Brien, Rahul Khosla, Jeremy S. Boyd, Brain Bales, Erin Wetherbee, Harald Sauthoff, Christopher K. Schott, Zahir Basrai, Dana Resop, Brian P. Lucas, Sandra Sanchez-Reilly, Sara Espinosa, Nilam J. Soni, Robert Nathanson

**Affiliations:** 1 South Texas Veterans Health Care System, Department of Geriatrics, Gerontology and Palliative Medicine, San Antonio, TX USA; 2 Division of Hospital Medicine, Emory School of Medicine, Atlanta, GA USA; 3 Medicine Service, Atlanta VA Medical Center, Atlanta, GA USA; 4 South Texas Veterans Health Care System, Research Service, San Antonio, TX USA; 5 Louis Stokes Cleveland VA Medical Center, Cleveland, OH USA; 6 Medicine Service, South Texas Veterans Health Care System, San Antonio, TX USA; 7 Division of Pulmonary Diseases & Critical Care Medicine, University of Texas Health San Antonio, San Antonio, TX USA; 8 Health Analysis and Information Group, Department of Veterans Affairs, Milwaukee, WI USA; 9 Pulmonary and Critical Care Medicine, Veterans Affairs Medical Center, Washington, DC USA; 10 Department of Pulmonary, Critical Care and Sleep Medicine, The George Washington University, Washington, DC USA; 11 Department of Emergency Medicine, VA Tennessee Valley Healthcare System—Nashville, Nashville, TN USA; 12 Department of Emergency Medicine, Vanderbilt University Medical Center, Nashville, TN USA; 13 Pulmonary Section, Minneapolis Veterans Affairs Health Care System, Minneapolis, MN USA; 14 Division of Pulmonary, Allergy, Critical Care and Sleep Medicine, Department of Medicine, University of Minnesota, Minneapolis, MN USA; 15 Medicine Service, VA NY Harbor Healthcare System, New York, USA; 16 Division of Pulmonary, Critical Care, and Sleep Medicine, New York University School of Medicine, New York, NY USA; 17 Critical Care Service, VA Pittsburgh Health Care Systems, Pittsburgh, PA USA; 18 Departments of Critical Care Medicine and Emergency Medicine, University of Pittsburgh, Pittsburgh, PA USA; 19 Emergency Medicine, VA Greater Los Angeles Healthcare System, Los Angeles, CA USA; 20 Department of Emergency Medicine, David Geffen School of Medicine at UCLA, Los Angeles, CA USA; 21 Department of Emergency Medicine, University of Wisconsin, Madison, WI USA; 22 Emergency Department, William S. Middleton Memorial Veterans Hospital, Madison, WI USA; 23 Medicine Service, White River Junction VA Medical Center, White River Junction, VT USA; 24 Department of Medicine, Dartmouth Geisel School of Medicine, Hanover, NH USA; 25 Division of Hospital Medicine, University of Texas Health San Antonio, San Antonio, TX USA

**Keywords:** Geriatricians, Point-of-care ultrasound, Veterans administration

## Abstract

**Background:**

Point-of-care ultrasound (POCUS) can aid geriatricians in caring for complex, older patients. Currently, there is limited literature on POCUS use by geriatricians. We conducted a national survey to assess current POCUS use, training desired, and barriers among Geriatrics and Extended Care (“geriatric”) clinics at Veterans Affairs Medical Centers (VAMCs).

**Methods:**

We conducted a prospective observational study of all VAMCs between August 2019 and March 2020 using a web-based survey sent to all VAMC Chiefs of Staff and Chiefs of geriatric clinics.

**Results:**

All Chiefs of Staff (*n*=130) completed the survey (100% response rate). Chiefs of geriatric clinics (“chiefs”) at 76 VAMCs were surveyed and 52 completed the survey (68% response rate). Geriatric clinics were located throughout the United States, mostly at high-complexity, urban VAMCs. Only 15% of chiefs responded that there was some POCUS usage in their geriatric clinic, but more than 60% of chiefs would support the implementation of POCUS use. The most common POCUS applications used in geriatric clinics were the evaluation of the bladder and urinary obstruction. Barriers to POCUS use included a lack of trained providers (56%), ultrasound equipment (50%), and funding for training (35%). Additionally, chiefs reported time utilization, clinical indications, and low patient census as barriers.

**Conclusions:**

POCUS has several potential applications for clinicians caring for geriatric patients. Though only 15% of geriatric clinics at VAMCs currently use POCUS, most geriatric chiefs would support implementing POCUS use as a diagnostic tool. The greatest barriers to POCUS implementation in geriatric clinics were a lack of training and ultrasound equipment. Addressing these barriers systematically can facilitate implementation of POCUS use into practice and permit assessment of the impact of POCUS on geriatric care in the future.

**Supplementary Information:**

The online version contains supplementary material available at 10.1186/s12877-023-04313-2.

## Background

Geriatric patients can be medically complex due to their multimorbidity, polypharmacy, frailty, disability, and social hardship [1]. The increasing availability of affordable handheld point-of-care ultrasound (POCUS) devices can enhance clinical decision-making and guide the care of complex geriatric patients [2, 3]. POCUS allows clinicians to rapidly rule in or rule out medical conditions, particularly urgent or emergent conditions, and may be an additional source of clinical revenue for geriatricians. In heart failure patients, randomized studies have shown the use of lung ultrasound to guide diuresis can reduce urgent care visits and rehospitalizations for heart failure [4–7]. During home visits, POCUS use can decrease the need for patient transportation for comprehensive diagnostic imaging and may improve patient experience [8]. Several studies have demonstrated the utility of measuring thigh muscle thickness by ultrasound for assessing frailty of geriatric patients in ambulatory, preoperative, and emergency settings [9–11].

More medical schools and internal medicine residency training programs are providing POCUS training, [12–15] but POCUS is not yet required by the Accreditation Council for Graduate Medical Education (ACGME) for internal medicine residency or geriatrics fellowship [16]. In a study of geriatric fellows, all fellows expressed a strong desire to learn how to use POCUS in their clinical practice [17]. Despite the growing interest in POCUS training, little is known about current POCUS use among attending physicians practicing geriatrics [18].

To better understand POCUS usage among geriatricians, we conducted a national survey to assess current use, training desired, and barriers to POCUS use among geriatricians practicing in Geriatrics and Extended Care (“geriatric”) clinics in the Department of Veterans Affairs (VA). Our findings can have important clinical implications for systematic implementation of POCUS use and training in geriatrics as a specialty.

## Methods

We performed a prospective observational study of all VA medical centers from June 2019 to March 2020. A multidisciplinary POCUS Technical Advisory Group with physicians from emergency medicine, internal medicine, hospital medicine, and critical care collaborated with the VA’s Healthcare Analysis and Information Group to develop and disseminate a web-based survey system-wide (Verint Systems, Inc.® 2019). This study was reviewed by the Investigational Review Board of the University of Texas Health Science Center San Antonio and deemed to be non-research (Protocol Number: HSC20210630NRR).

The web-based survey included questions on current use, barriers to use, institutional support, equipment, and training needs of POCUS [19–22]. Question types were multiple-choice; forced-choice (yes/no); open-ended with numerical or free text entry; and free text boxes when “other” was selected.

The survey was deployed in two phases. First, a survey was distributed to all Chiefs of Staff (*n* = 130) who oversee all clinical specialties at VA medical centers, similar to a chief medical officer [19–22]. The Chief of Staff survey included 10 questions about facility-level POCUS use, training, competency, and policies, and gathered contact information of all geriatric chiefs at a facility. Second, an 18-question follow-up survey was sent to all geriatric chiefs (“chiefs”) (n=76) identified by the Chiefs of Staff to collect data specifically about POCUS use in geriatrics. Chiefs reported service-level data on diagnostic and procedural POCUS use, training needs, workflows, and equipment availability on behalf of their geriatricians. The survey period for chiefs started in December 2019 but ended early in March 2020 due to the Covid-19 pandemic.

The Veterans Health Administration Service Support Center identified 76 VAMCs as having dedicated geriatric clinics, specializing in men and women veterans aged ≥ 65 years. The VA also has many community-based outpatient clinics located in facilities that are supervised independently of the local VAMCs, and a few of these outpatient clinics have dedicated geriatric teams. However, these geriatric teams at community-based outpatient clinics were not included in this survey.

## Results

**Fig. 1:**
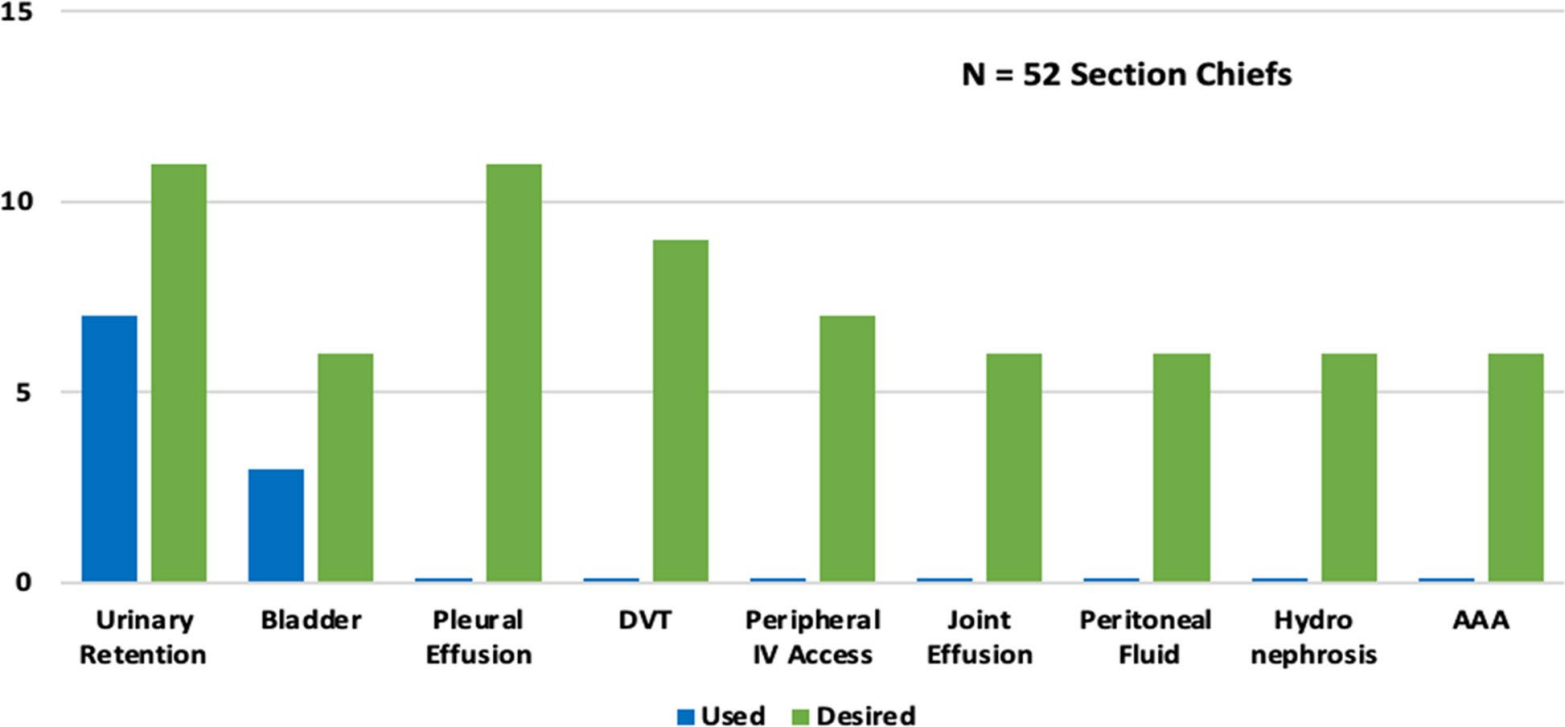
Most Common POCUS Applications Used and Training Desired in Geriatric Care. The survey had 68 applications including cardiac, pulmonary, abdominal, procedures, skin/soft tissues/musculoskeletal, and other systems. POCUS, point-of-care ultrasound; DVT, deep vein thrombosis, IV, intravenous; AAA, abdominal aortic aneurysm

All Chiefs of Staff (*n* = 130) completed the survey (100% response rate). Seventy-six chiefs of geriatric clinics at different VAMCs were surveyed, and 52 responses were received for a response rate of 68%. Survey responses on current use and training of POCUS from the Chiefs of Staff from these 52 facilities are included in Supplementary tables. Most geriatric clinics reported caring for high-complexity patients in an urban setting. Only 15% of geriatric clinics reported having ≥ 1 provider using POCUS (Table 1). The most common POCUS applications used in geriatric clinics were urinary retention (13%) and bladder exams (6%). Chiefs reported a wide range of applications for which they desired training (Fig. 1). Though 25% of geriatric clinic chiefs reported a desire for POCUS training, only 27% had a process in place for providers to obtain POCUS training. However, more than 60% of chiefs would support a local or regional POCUS course to train their geriatricians. One-fourth of chiefs were aware of specific policies in place at their VAMC related to POCUS use, such as credentialing, machine maintenance, and documentation of findings (Table 1).

**Table 1:** Characteristics and Current use of POCUS in Geriatric Clinics at VA Medical Centers (*N* = 52 Facilities)

Characteristic	Data
**Active Geriatric Patients (2019-2020)**	
<500	25 (48%)
500–1,500	18 (35%)
>1,500	9 (17%)
**VA Facility Complexity Level**^a﻿^	
High	46 (88%)
Low	6 (12%)
**Region**	
Northeast	11 (21%)
Midwest	13 (25%)
South	19 (37%)
West	9 (17%)
**Location**	
Urban	51 (98%)
**Current Use**	
At least one Geriatric provider uses POCUS	8 (15%)
Providers have desire for POCUS Training	13 (25%)
Service Chief knows of ≥1 facility-wide policy for POCUS	14 (27%)
Current process to obtain POCUS training	14 (27%)
Service Chief supports POCUS training	32 (62%)

^a^High-complexity facilities have high levels of patient volume, patient risk, specialists, teaching, and research. Low-complexity facilities have medium to low patient volume levels and risk levels, and some to little teaching or research. POCUS, point-of-care ultrasound; VA, Veterans Affairs

Barriers to POCUS use were categorized as training, equipment, or infrastructure (Table 2). The most common barrier reported was a lack of trained providers (56%). Further, 35% of chiefs reported a lack of funding for training, 27% reported a lack of training opportunities, and 23% felt there was a lack of funding for travel to receive training. The second most common barrier was the lack of ultrasound equipment which was reported by 50% of geriatric chiefs. Approximately one-quarter of chiefs reported that they perceived little or no benefit from POCUS use.

**Table 2:** Barriers to POCUS use Among Geriatric and Extended Care Clinics per Geriatric Chiefs and Chiefs of Staff

Barriers	Geriatric Chiefs Reporting Barriers (*N*=52)	Chiefs of Staff Reporting Barriers (*N*=52)
**TRAINING**		
Lack of Trained Providers	29 (56%)	33 (63%)
Lack of Funding for Training	18 (35%)	21 (40%)
Lack of Training Opportunities	14 (27%)	26 (50%)
Lack of Funding for Travel	12 (23%)	13 (26%)
One or More TRAINING Barriers Listed Above	30 (58%)	41 (79%)
**EQUIPMENT**		
Lack of Ultrasound Equipment	26 (50%)	25 (48%)
Lack of Funding for Ultrasound Equipment	11 (21%)	16 (31%)
One or More EQUIPMENT Barriers Listed Above	26 (50%)	27 (52%)
**INFRASTRUCTURE**		
No Clinician Champion	12 (23%)	12 (23%)
Lack of Funding for Support Staff	13 (25%)	16 (31%)
Lack of Funding for Simulation Space	9 (17%)	14 (27%)
Lack of Facility Leadership Support	6 (12%)	1 (2%)
Lack of Privileging Criteria	4 (8%)	10 (19%)
Lack of Standard Reporting Form	2 (4%)	12 ((23%)
Lack of Image Archiving	2 (4%)	19 (37%)
One or More INFRASTRUCTURE Barriers Listed Above	21 (40%)	33 (63%)
**OTHER**		
No Perceived Benefit	13 (25%)	3 (6%)
No Barriers Identified	9 (17%)	8 (15%)

Qualitative analysis of open-ended questions revealed both favorable and unfavorable comments toward POCUS use in geriatric clinics. Some chiefs (*n* = 6) believed that POCUS has limited utility in geriatric clinics, especially at large, urban VAMCs where imaging and consultative services are readily available. Five chiefs commented that any patient needing a POCUS exam would be sent to the emergency department, radiology, or inpatient medicine service for further evaluation. Though some chiefs (*n* = 3) recognized the potential benefits, they stated lack of training and infrastructure would preclude POCUS implementation in geriatric clinics. Three chiefs felt there was insufficient time during patient visits to perform POCUS exams.

## Discussion

We have conducted the largest systematic survey of POCUS use by geriatricians, and our findings can guide the implementation of POCUS use in geriatric clinics. A minority of chiefs reported current POCUS use within their geriatric clinics, but most recognized the potential benefits of POCUS use, would support training their geriatricians through a local or regional course, and identified key barriers, such as lack of training, that must be addressed to promote adoption of POCUS.

### Current use and training

POCUS has shown promise in several specialties including geriatrics and primary care [8, 17, 23–25]. POCUS can increase the diagnostic yield of routine examinations and readily detect conditions that are prevalent in elderly patients, including cardiac disease, acute respiratory illnesses, abdominal aortic aneurysm, and urinary retention [25–27]. Despite the potential benefits and growth of POCUS training in internal medicine residency programs, [12] a recent retrospective review of Medicare Part B claims data showed that geriatricians comprised only a small portion of POCUS users [28]. Our study confirmed that relatively few geriatricians are currently using POCUS.

We have described the POCUS applications that geriatricians currently use and for which they desire training. These results may inform future curricula development. Currently, the most commonly used applications were evaluation of the bladder and urinary retention which are common indications in the geriatric population [8]. Chiefs also reported a desire for training in lung, deep venous thrombosis, musculoskeletal, and abdominal aortic applications. Emerging evidence exists for use of these applications in geriatric and primary care settings [23, 27]. Surprisingly, few chiefs reported current use or desire for training in cardiac ultrasound in our study. However, in studies of geriatric fellows and geriatricians in home-based primary care settings, cardiac ultrasound for volume status assessment was a commonly performed and highly desired application due to the provision of real-time clinical information that frequently altered management [8, 17]. Furthermore, given the high prevalence of cardiovascular disease in the elderly, POCUS may also serve as a screening tool for geriatricians to detect occult changes in cardiac function [3].

As illustrated in the open-ended survey responses, many chiefs may be unaware of all the POCUS applications and their potential benefits in geriatrics [9, 10, 23, 25]. Most chiefs support POCUS training for geriatricians in their practice[17, 18]. Future development of curricular guidelines and competency standards for geriatricians is needed, [17] as well as further research on outcomes of POCUS-guided care in geriatrics [9, 10].

### Barriers

We have highlighted important barriers to POCUS use in geriatrics. The lack of trained providers was the most commonly reported barrier. Although more medical schools and internal medicine residency programs are incorporating POCUS into training curricula, few geriatric fellowships currently provide POCUS training [12, 17]. Training-related barriers, including lack of POCUS experts to oversee training and time for practice, are well described barriers to POCUS use in multiple specialties in the United States [19–21, 29, 30] and other countries [31, 32]. Therefore, development of national POCUS training programs for academic geriatricians are needed to train geriatricians in-practice and ensure adequate supervision of trainees.

Lack of available ultrasound equipment was another prominent barrier reported by half of geriatric chiefs. Access to ultrasound machines is essential to improve utilization and workflow efficiency, as well as achieve and maintain competency [33]. Handheld ultrasound devices have become increasingly available and may serve as alternatives to cart-based ultrasound machines given their lower cost, greater portability, and comparable sensitivity and specificity for common diagnostic POCUS applications [2]. Incorporation of handheld ultrasound devices may enhance clinical decision-making and improve quality and timeliness of care in home-based primary care and hospital-in-home settings [8, 34].

Other key barriers revealed by our study include time constraints and lack of program infrastructure. Time constraints during busy medical encounters with complex geriatric patients and administrative burdens of documenting and billing for POCUS exams have been previously reported [17, 18, 28]. Restructuring patient encounters with physicians obtaining a medical history simultaneously while performing a POCUS examination may improve efficiency and shared diagnostic understanding with patients [35, 36]. Investment in program infrastructure, including image archiving systems and documentation templates, is necessary to promote widespread, standardized adoption of POCUS use in geriatrics. Similar to other specialties, geriatrics will need to discuss and gather consensus about POCUS use in clinical care and the minimum infrastructure needed for POCUS implementation.

### Strengths and limitations

Our study had a high response rate and collected data systematically from geriatric practices at VAMCs nationwide. Limitations include the collection of self-reported data from geriatric chiefs which may not accurately reflect actual clinical practice. Further, findings may not be generalizable to providers practicing outside of geriatric VAMC clinics, although many VAMCs are staffed by providers who practice at affiliated medical schools and non-VA facilities. Further, our survey did not assess current POCUS use in geriatric clinics located outside of VAMCs, including home-based primary care and hospital-in-home settings. Finally, our data are limited to the United States and may not be relevant to healthcare systems in Europe or other countries.

## Conclusions

POCUS has many potential benefits for the management of complex geriatric patients with multimorbidity. Currently, few geriatric clinics are using POCUS. The desire for training exceeds current use, and most geriatric chiefs would support POCUS training for their geriatricians. Barriers to implementation of POCUS use in geriatric clinics included lack of trained providers and ultrasound equipment, as well as time constraints. To support expanded POCUS use by geriatricians, development of standardized curricula and investment in training, ultrasound equipment, and program infrastructure is needed.

### Supplementary Information

**Additional file 1: Table S1.** Desire for POCUS Training at VAMCs per COSs (*N*=52). **Table S2.** Established Policies on POCUS use at VAMCs per COSs (*N*=52). **Table S3.** Competency Standards for Providers using POCUS at VAMC per COSs (*N*=52). **Table S4.** Training Support & Availability at VAMC per COSs (*N*=52)

## Data Availability

The data supporting the findings of this study are available within the article.

## Abbreviations

POCUS: Point-of-care ultrasound
Geriatric clinics: Geriatrics and Extended Care clinics
VAMCs: Veterans Affairs Medical Centers
Chiefs: Chiefs of geriatric clinics
ACGME: Accreditation Council for Graduate Medical Education
VA: Department of Veterans Affairs
