# Supplementary material for: Point-of-care ultrasound in geriatrics: a national survey of VA medical centers
Source: BMC Geriatr. 2023 Sep 27;23:605. doi: 10.1186/s12877-023-04313-2 (PMC10537073; doi:10.1186/s12877-023-04313-2)
Supplement: Supplementary file 1 — Additional file 1: Table S1. Desire for POCUS Training at VAMCs per COSs (N=52). Table S2. Established Policies on POCUS use at VAMCs per COSs (N=52). Table S3. Competency Standards for Providers using POCUS at VAMC per COSs (N=52). Table S4. Training Support & Availability at VAMC per COSs (N=52) [file 12877_2023_4313_MOESM1_ESM.docx]

**Supplementary Tables**

(*Survey Responses from Chiefs of Staff (COSs) on Point-of-care Ultrasound (POCUS) use at Veterans Affairs Medical Centers (VAMCs) with Responses from Geriatrics Chiefs)*

**Table S1: Desire for POCUS Training at VAMCs per COSs (N=52)**

| **POCUS Application** | **Number of COSs Endorsing Use** |
| --- | --- |
| Peripheral IV Access | 33 (63%) |
| Urinary Retention | 35 (67%) |
| Bladder | 25 (48%) |
| Other Abdominal Applications | 42 (81%) |
| Pulmonary Applications | 40 (77%) |
| Cardiac Applications | 38 (73%) |
| Musculoskeletal Applications | 38 (73%) |
| Deep Vein Thrombosis | 34 (65%) |
| No Applications Endorsed | 9 (17%) |

IV, intravenous.

**Table S2: Established Policies on POCUS use at VAMCs per COSs (N=52)**

| **Facility-wide Policy Type** | **Number of COSs Reporting** |
| --- | --- |
| No POCUS policies | 40 (77%) |
| Use by Clinicians | 5 (10%) |
| Ultrasound Equipment Maintenance | 8 (15%) |
| Documentation/Image Archiving | 4 ( 8%) |
| Privileging of Clinicians | 7 (13%) |
| Supervision of Trainees | 7 (13%) |

**Table S3: Competency Standards for Providers using POCUS at VAMC per COSs (N=52)**

| **Characteristics** | **Number of COS Reporting** |
| --- | --- |
| Facility has formal credentialing/privileging process to perform specific POCUS exams | 15 (29%) |
| Facility requires initial demonstration of provider competency in POCUS to grant privileges | 20 (38%) |
| ***Initial competency methods used:***  Attestation of competency obtained during GME  Accredited POCUS CME with hands-on sessions  Demonstrated/Tracked at academic affiliate  Documented minimum number of cases  Focused Professional Practice Evaluation  Live Proctoring  Simulation demonstration | 18 (35%)  13 (25%)  6 (12%)  7 (13%)  16 (31%)  9 (17%)  3 (6%) |
| Facility requires ongoing demonstration of provider competency in POCUS to maintain privileges | 15 (29%) |
| ***Ongoing competency methods used:***  Attestation of competency from supervisor  Personal attestation  Completed/Tracked at academic affiliate  Documented minimum number of cases since last assessment  Ongoing Professional Practice Evaluation  Live Proctoring  Simulation demonstration | 11 (21%)  1 (2%)  1 (2%)    4 (8%)  10 (19%)  2 (4%)  1 (2%) |

GME, graduate medical education; CME, continuing medical education.

**Table S4: Training Support & Availability at VAMC per COSs (N=52)**

| **Training Opportunity** | **Number of COSs Reporting** |
| --- | --- |
| COS in favor of physicians attending national or regional VA POCUS course | 42 (81%) |
| COS would support onsite POCUS training course by VA, if necessary, resources provided by trainers | 45 (87%) |
| **Training opportunities that currently exist locally**:  Onsite CME  Offsite CME sponsored by VA  Non-VA sponsored (e.g., academic affiliation) | 6 (12%)  9 (17%)  25 (48%) |

CME, continuing medical education; VA, Veterans Affairs.
